# Antimicrobial and Antibiofilm Activity of Monolaurin against Methicillin-Resistant *Staphylococcus aureus* Isolated from Wound Infections

**DOI:** 10.1155/2024/7518368

**Published:** 2024-08-02

**Authors:** Shimaa Salah Hassan Abd El-Ghany, Ahmed Farag Azmy, Ahmed Osama EL-Gendy, Rehab Mahmoud Abd El-Baky, Ahmad Mustafa, Mohammed A. S. Abourehab, Mohamed E. El‐Beeh, Reham Ali Ibrahem

**Affiliations:** ^1^ Department of Microbiology and Immunology Faculty of Pharmacy Beni-Suef University, Beni-Suef 62514, Egypt; ^2^ Department of Microbiology and Immunology Faculty of Pharmacy Deraya University, Minia 11566, Egypt; ^3^ Department of Microbiology and Immunology Faculty of Pharmacy Minia University, Minia 61519, Egypt; ^4^ Faculty of Engineering October University for Modern Science and Arts (MSA), Giza, Egypt; ^5^ Department of Pharmaceutics Faculty of Pharmacy Minia University, Minia 61519, Egypt; ^6^ Department of Pharmaceutics Faculty of Pharmacy Umm Al-Qura University, Makkah 21955, Saudi Arabia; ^7^ Biology Department Al‐Jumum University College Umm Al‐Qura University, Makkah 21955, Saudi Arabia

## Abstract

**Background:**

Methicillin-resistant *Staphylococcus aureus* (MRSA) is one of the major pathogens associated with life-threatening infections, showing resistance to various antibiotics. This study aimed to assess the influence of monolaurin on biofilm-forming MRSA.

**Methods:**

The agar dilution method determined the minimum inhibitory concentration (MIC) of monolaurin against MRSA isolates and explored its impact on the resistance profile of selected antibiotics. The assessment of combined therapy involving monolaurin and antibiotics was conducted using fractional inhibitory concentration (FIC). The tissue culture plate strategy appraised monolaurin's antibiofilm activity and its inhibitory concentration (IC_50_), with assessment via scanning electron microscopy. Reverse transcription polymerase chain reaction (RT-PCR) discerned a monolaurin effect on the expression of the *icaD* gene.

**Results:**

Monolaurin exhibited MIC values ranging from 500 to 2000 *μ*g/mL. FIC index showed a synergistic effect of monolaurin with *β*-lactam antibiotics ranging from 0.0039 to 0.25 (*p* < 0.001). Among the 103 investigated MRSA isolates, 44 (44.7%) displayed moderate biofilm formation, while 59 (55.3%) were strong biofilm producers. Antibiofilm activity demonstrated concentration dependence, confirming monolaurin's capacity to inhibit biofilm formation and exhibited strong eradicating effects against preformed MRSA biofilms with IC_50_ values of 203.6 *μ*g/mL and 379.3 *μ*g/mL, respectively. Scanning electron microscope analysis revealed reduced cell attachments and diminished biofilm formation compared to the control. The expression levels of the *icaD* gene were remarkably reduced at monolaurin concentrations of 250 and 500 *μ*g/mL.

**Conclusion:**

Monolaurin had significant inhibitory effects on MRSA pre-existing biofilms as well as biofilm development. So, it can be employed in the treatment of severe infections, particularly those associated with biofilm formation including catheter-associated infections.

## 1. Introduction

The emergence of infections associated with multidrug-resistant (MDR) microorganisms is a serious global issue and a great challenge for healthcare teams worldwide [1]. Over recent years, there has been a notable increase in the incidence of *Staphylococcus aureus* infections, especially those infections caused by MDR *Staphylococci* that exhibit heightened morbidity and mortality compared to infections caused by other bacteria [2]. Infections with *S. aureus* can range from minor dermatological infections to major illnesses such as septicemia, toxin-mediated toxic shock syndrome, endocarditis, pneumonia, and osteomyelitis. Besides, it can cause chronic skin infections and mastitis in farm animals [3, 4]. The threat posed by *S. aureus* infections is increased by the emergence of methicillin-resistant *S. aureus* (MRSA) and nonspecific resistance mechanisms including biofilms. The development of biofilms on either host tissues, such as implanted materials, including dialysis catheters, orthopedic implants, prosthetic heart valves, urine catheters, and central venous catheters, has been implicated in many of these pathological conditions [5, 6]. A considerable proportion of infections, those associated with embedded medical devices, was thought to be caused by microbial biofilms, as indicated by various estimations [7]. Microbial biofilms are ubiquitous as the predominant life forms for microbes across various systems [8]. Bacterial biofilms are complex communities encased within a self-produced extracellular matrix primarily composed of polysaccharides, proteins, lipids, and extracellular DNA (eDNA). This matrix plays a crucial role in protecting biofilm bacteria from host immune responses. Biofilm bacteria display emergent properties distinct from free-living cells, including enhanced genetic exchange, formation of synergistic microconsortia, and increased resistance to antimicrobials, attributed largely to the EPS matrix [9, 10]. In addition, biofilms facilitate the spread of antibiotic resistance through horizontal gene transfer. However, detecting bacterial biofilms presents challenges due to their inherent tolerance to host defenses and conventional antibiotic treatments, complicating routine diagnostic procedures [11]. The treatment choices were also limited as a result of these biofilm's high resistance to host defenses and antimicrobial treatments [6]. The therapeutic management of MRSA in Egypt had encountered a considerable difficulty with a concerning increase in prevalence over time necessitating efficient microbial control strategies that disrupt the antibiotic resistance cycle [12]. Thus, the imperative to find biofilm inhibitors arises as a critical need from this circumstance.

Monolaurin is a byproduct of coconut oil, prepared from lauric acid and glycine. So, glycerol monolaurate (GML) is a fatty acid monoester. The Food and Drug Administration (FDA) recognizes glycerol monolaurate (GML) as a safe, natural substance that is frequently used as an additive in cosmetic and food manufacturing. In addition, GML exhibits broad-spectrum antibacterial properties, particularly against Gram-positive bacteria including Gram-positive cocci. Qanash et al. [13] reported the antimicrobial efficacy of coconut oil nanoemulsion (NE) against some microorganisms including *S. aureus* and their ability to decrease inflammation. Also, Khan et al. [14] elucidated GML's potential in reducing biofilm formation and as an antiquorum sensing agent. Monolaurin probably uses several different processes to work, though. Also, GML inhibits the generation of Gram-positive exotoxins and eliminates pre-existing *S. aureus* biofilms [15]. The use of monolaurin, which precisely targets key stages involved in biofilm formation and has shown antimicrobial activity, would be a prudent approach for lowering the risk of infections caused by *S. aureus* biofilms. Therefore, our study was conducted to assess the antimicrobial resistance patterns of MRSA strains, evaluate the potential impact of monolaurin on the antimicrobial susceptibility of the isolated MRSA strains, and ascertain its influence on biofilm formation and preformed biofilm produced by the tested isolates.

## 2. Materials and Methods

### 2.1. Materials

Monolaurin 99% was provided by Oleo Misr for Oleochemicals Company, Sadat city, Egypt.

### 2.2. Study Area, Design, and Population

A cross-sectional observational study was carried out from September 2021 to April 2022 at Minia University Hospital (Minia, Egypt). A total of 155 clinical specimens were obtained from patients who were admitted to Minia University hospitals in Egypt with wound infections. Being part of standard hospital laboratory protocols, the clinical samples were collected and labeled with the patient's name and the source. The Ethics Committee of the Deraya University's faculty of pharmacy approved the study's use of human participants.

### 2.3. Inclusion and Exclusion Criteria

The inclusion criteria comprised patients meeting the following conditions: (a) according to the following wound etiologies: surgical wounds, accidental wounds, abscesses, ulcers, and burns with positive (+) bacterial culture results; (b) age between 1 and 60 years; and (c) provision of informed consent by the patients. Conversely, the exclusion criteria encompassed the following: (a) patients exhibiting severe dysfunction of vital organs such as the heart, brain, liver, kidneys, or other major organs or suffering from severe primary diseases; (b) patients with underlying medical conditions impacting wound healing, including hematologic disorders, immune system disorders, and connective tissue diseases; (c) patients concurrently participating in other clinical studies; and (d) patients demonstrating inadequate compliance.

### 2.4. Bacterial Investigation

Samples were cultured on brain heart infusion broth at 37°C for 24 hours. For isolation and purification, mannitol salt agar was streaked with a loopful of bacterial suspension and incubated for 24 hours at 37°C. *S. aureus* isolates were initially recognized through the appearance, fermentation of mannitol sugar, morphology, gram staining, and biochemical characteristics of the colony (tests for catalase, coagulase (tube and slide), and DNase).

### 2.5. Phenotypic Identification of MRSA and Antimicrobial Susceptibility Testing

#### 2.5.1. Screening of Methicillin-Resistant *S. aureus*

Methicillin-resistant *S. aureus* isolates were identified using cefoxitin discs (30 *μ*g) on Mueller–Hinton agar plates according to Clinical and Laboratory Standards Institute guidelines (CLSI, 2020). The criteria for MRSA categorization were based on the measurement of inhibition zones, with isolates exhibiting a diameter of zones ≤21 mm considered resistant and thus classified as MRSA [16].

#### 2.5.2. Antimicrobial Susceptibility Testing

All MRSA strains were subjected to antimicrobial susceptibility testing against ampicillin/sulbactam (20 *μ*g), amoxicillin/clavulanic acid (30 *μ*g), piperacillin/tazobactam (10 *μ*g), gentamicin (10 *μ*g), amikacin (30 *μ*g), ciprofloxacin (5 *μ*g), levofloxacin (5 *μ*g), tetracycline (30 *μ*g), chloramphenicol (30 *μ*g), imipenem (10 *μ*g), rifampicin (5 *μ*g), and linezolid (30 *μ*g) (Oxoid, UK) [16]. Following Magiorakos et al. [17], vancomycin susceptibility of isolates was evaluated using the agar dilution method.

### 2.6. Effect of Monolaurin on the Growth of Tested Isolates, Activity of Some Antibiotics, and Biofilm Formation

#### 2.6.1. Effect of Monolaurin on the Growth of MDR MRSA

*Determination of Minimum Inhibitory Concentration (MIC) of Monolaurin against the Tested Isolates*. The agar dilution method was employed for MDR MRSA isolates (*n* = 103) to determine the MICs for monolaurin. The tested isolates were cultured overnight in Mueller–Hinton broth (MHB), with a cell density of 10^7^ colony-forming units per milliliter (CFU/mL) of cells. Successive two-fold dilutions of monolaurin ranging from 15.625 to 2000 *μ*g/mL were added to Mueller–Hinton agar (MHA). The application of microbial inoculum to the agar plate's surface was executed using a multi-inoculator. The plates were incubated for 24 h at 37°C [18, 19].

#### 2.6.2. Evaluating the Impact of Monolaurin and Specific *β*-Lactam Antibiotics Combinations through Fractional Inhibitory Concentration (FIC) Assay

To test the synergy, the agar dilution method was used. The FIC assay was performed to assess the effectiveness of monolaurin in combination with the examined antibiotics at sub-MIC doses against MDR MRSA isolates. The antibacterial interactions were categorized into antagonistic (FICi > 2), indifferent (0.75 < FICi ≤ 2), partial synergistic (0.5 < FICi ≤ 0.75), or total synergistic (FICi ≤ 0.5) [20].

#### 2.6.3. Effect of Monolaurin on Biofilm Formation

*(1) Biofilm Detection using Tissue Culture Plate (TCP) Method*. According to Manandhar et al. [21], bacterial culture was cultivated at 37°C for 24 h in tryptic soy broth (TSB) supplied with 1% (w/v) glucose, followed by a 1 : 100 dilution using fresh medium. Next, the diluted culture (200 *μ*L) was dispensed into individual wells of a 96-well microtiter plate. Negative controls included uninoculated tryptic soy broth which was incubated for 48 h for the plates at 37°C. Then, the contents of each well were taken out, and the wells were washed three times with 200 *μ*L of sterile, distilled water. Adherent biofilms were first fixed for 15 minutes with 95% ethanol and then stained for 5 minutes with 100 *μ*L of 1% crystal violet. After eliminating unbound dye, the wells were rinsed with 200 *μ*L of sterile distilled water and air-dried. To dissolve the formed biofilm, 100 *μ*L of ethanol was applied. Finally, the optical densities (ODs) of stained adherent biofilms were quantified at a wavelength of 570 nm using a micro-ELISA auto reader. Three duplicates of the experiment were conducted. The average OD values of the sterile medium were omitted from all test results once they had been calculated. ODs below 0.120 were considered nonbiofilm producers, ODs between 0.120 and 0.240 were considered moderate biofilm producers, and ODs greater than 0.240 were considered strong biofilm producers [22, 23].

*(2) Measuring the Effectiveness of Different Monolaurin Concentrations in Eradicating Biofilms*. The efficacy of monolaurin in disrupting preformed biofilms was investigated. A suspension of the tested strains (100 *μ*L) was inoculated into individual wells of a 96-well tissue culture plate. Uninoculated TSB was considered as a negative control. Incubation of plates was at 37°C for 24 h. Wells were then washed with 200 *μ*L of phosphate buffer saline (PBS). Then, 100 *μ*L of each tested concentration of monolaurin (250–2000 *μ*g/mL) was added to each well. Normal saline was added to control wells (positive control and negative control). Plates were incubated at 37°C for 24 h. Supernatants were cast off, and the wells were washed at least twice by 200 *μ*L of PBS. Biofilm was measured using crystal violet assay. At 570 nm, the biofilm's OD was determined after a 24-hour aerobic incubation period at 37°C. The mean biofilm OD at the used concentrations of monolaurin was found to be equal to or less than the OD of the negative control. The percentage of inhibition of preformed biofilm was calculated by using the following formula:

(1) $$\begin{array}{c} P\text{ercentage of inhibition }\left(\%\right)=\frac{\text{OD}\left(\text{Negative Control}\right)-\text{OD}\left(E\text{xperimental}\right)}{\text{OD}\left(\text{Negative Control}\right)}\times 100. \end{array}$$

The activity of monolaurin on the preformed biofilm against all MRSA isolates was expressed as the 50% inhibitory concentration (IC_50_). The percentage of inhibition was plotted against monolaurin concentration using Prism software (GraphPad Prism 9 software) to determine the IC_50_ value [24, 25]. Trials were conducted in triplicate [26].

*(3) Measuring the Effectiveness of Different Monolaurin Concentrations on Biofilm Formation*. Monolaurin's capability to inhibit biofilm formation was assessed. The bacterial suspension (100 *μ*L) was distributed in tissue culture plate wells. One hundred microliters of varying concentrations of monolaurin (250, 500, 1000, and 2000 *μ*g/mL) were added to the wells containing bacterial suspension, while biofilms were allowed to grow as previously mentioned. Plates were incubated at 37°C for 24 h. Supernatants were discarded, and wells were washed twice by PBS. The effect of monolaurin on biofilm formation was detected by crystal violet assay at 570 nm. As a positive control, MRSA isolate culture was used, while the negative control was uninoculated tryptic soy broth (Oxoid, USA). IC_50_ value of monolaurin activity on biofilm formation was calculated as previously mentioned. The experiments were performed three times [26].

### 2.7. Scanning Electron Microscope

Following treatment with monolaurin, biofilm development was observed using a scanning electron microscope (SEM). Monolaurin and TSB were combined at 1×MIC and 2×MIC of the tested strains and then pipetted onto sterile culture plates containing MRSA. Untreated cells were used as control. Every dish had a sterile plastic coverslip in it. Following the instructions of the Huazhong Agricultural University's microscopy core laboratory, the coverslips were carefully cleaned twice with sterile PBS and then 2.5% glutaraldehyde/PBS (v/v, pH 7.2) was applied. After that, the samples underwent ethanol dehydration. The coverslips were carefully cleaned twice with sterile PBS following an overnight incubation at 37°C. Using a Hitachi SU8010, MRSA isolates biofilm formation on the coverslips was detected [27].

### 2.8. Conventional PCR *icaA* and *icaD* Biofilm Genes

The procedures were carried out in accordance with the manufacturer's instructions upon using a DNA extraction kit (QIAamp DNA Mini Kit, Germany). For the *icaA* gene, the oligonucleotide primer sequences (Metabion, Germany) were as follows: forward primer 5′ CCT AAC TAA CGA AAG GTA G 3′ and reverse primer 5′ AAG ATA TAG CGA TAA GTG C 3′ with amplicon size 1315 bp, and *icaD* gene, F 5′AAA CGT AAG AGA GGT GG 3′ and R 5′ GGC AAT ATG ATC AAG ATA3′ with amplicon size 381 bp [28]. The conditions used to conduct the polymerase chain reaction (PCR) are summarized in Supplementary Table (ST-1). Electrophoresis using 1.5% agarose gel for 30 minutes at a continuous current of 1–5 volts/cm was the applied technique for PCR product detection. DNA bands were identified by ethidium bromide staining and UV transillumination light.

### 2.9. Real-Time Polymerase Chain Reaction (RT-PCR)

RT-PCR was performed to measure the relative expression of the *icaD* gene in the absence and presence of 0.25×MIC and 0.5×MIC of monolaurin (Supplementary Table ST-2 contains a list of the primer sequences). Each experimental and control tube was incubated at 37°C for 24 h. The RNeasy Mini Kit's instructions were carried out to extract total RNA. The cycling conditions are shown in Supplementary Table (ST-3). Gene expression levels were standardized to 16S rRNA. Amplification curves and cycle threshold (CT) were computed using the start gene Mx3005P program. The CT of each sample was compared with that of the control, and the variation in gene expression among RNA samples was assessed using the “Ct” approach proposed by Yuan et al. [29]. Dissociation curves from several samples were evaluated to rule out false positive results.

### 2.10. Statistical Analysis

SPSS version 25.0 software was utilized for data analysis. To determine changes in the ODs of biofilms with increasing monolaurin concentrations, the general linear model for repeated measurements (for not normally distributed data) was employed. Using Wilcoxon's test, treated biofilms with various concentrations of monolaurin were compared with untreated biofilms (control).

## 3. Results

### 3.1. *Staphylococcus aureus* Isolation and Identification

A total of 155 samples from patients of different ages, who had been admitted to the Minia University hospitals in Egypt with wound infection between September 2021 and April 2022 were examined for bacterial growth. Clinical samples included the following: 63 samples from surgical wounds, 41 samples from accidental wound infections, 20 samples from abscesses and ulcers, and 31 samples from burns. By using several culture and biochemical techniques, 115 (74.19%) of these collected samples were identified as *S. aureus* bacterial isolates.

### 3.2. Prevalence of MRSA in Different Clinical Wound Samples

Within 115 *S. aureus,* 103 (89.56%) were MRSA. A higher incidence of MRSA isolates (37.9%) was recovered from accidental wound infection followed by surgical wounds (22.3%), burns (20.4%), and ulcers and abscesses (19.4%) (Supplementary Figure SF-1).

### 3.3. Resistance Pattern of MRSA Isolates

Regarding MRSA isolates, they exhibited complete resistance against ampicillin/sulbactam, amoxicillin/clavulanic acid, piperacillin/tazobactam, and cefoxitin (100%); moderate resistance against tetracycline (64.07%), rifampicin (40.77%), ciprofloxacin (38.83%), levofloxacin (38.83%), and gentamicin (37.86%); and low resistance against vancomycin (4.35%), imipenem (3%), and linezolid (2%) as illustrated in Supplementary Figure (SF-2). Based on the antimicrobial resistance patterns of these isolates, the MDR pattern is described in Supplementary Figure (SF-3).

### 3.4. Effect of Monolaurin on the Growth of Tested Isolates, Activity of Some Antibiotics, and Biofilm Formation

#### 3.4.1. Estimation of Minimum Inhibitory Concentration (MIC) of Monolaurin against Tested Isolates

MICs of monolaurin (glycerol monolaurate, GML) on MRSA clinical isolates are given in Table 1. The MIC values of monolaurin against MRSA ranged from 250 to 2000 *μ*g/mL, which indicates that using GML as a diet supplement in a concentration ranging from 1 to 5 g daily can inhibit microbial growth.

#### 3.4.2. Synergistic Effect of Monolaurin with the Tested Antibiotics

Clinical MRSA isolates were used to assess the combined effects of monolaurin with the various antibiotics (ampicillin, amoxicillin, and piperacillin). When accompanied with 250–500 *μ*g/mL of monolaurin, the MIC of ampicillin was reduced from 8–32 *μ*g/mL to 0.5–4 *μ*g/mL (*p* < 0.001) (Supplementary Table ST-4). Amoxicillin's MIC was 32–128 *μ*g/mL when tested individually. When combined with 250 and 500 *μ*g/mL of monolaurin, the MIC of amoxicillin was reduced from 0.5–8 to 0.5–4 *μ*g/mL (*p* < 0.001) (Supplementary Table ST-5). The MIC of piperacillin ranged from 16 to 256 *μ*g/mL when tested alone. In combination with 250–500 *μ*g/mL of monolaurin, the MIC of piperacillin decreased to 0.5–32 *μ*g/mL (*p* < 0.001) (Supplementary Table ST-6).

### 3.5. Detection of Biofilm-Forming MRSA Isolates

Assessment of biofilm formation was performed on the 103 MRSA isolates. According to the intensity of biofilm production, they were classified into two groups based on OD readings obtained from the microtiter plate assay. All MRSA isolates had various degrees of biofilm formation ability. About 59.28% of the isolates produced strong biofilms (*n* = 59), while 44.72% of them produced moderate biofilms (*n* = 44).

### 3.6. Antibiofilm Activity of Monolaurin on MRSA Isolates

#### 3.6.1. Monolaurin Activity against Preformed Biofilm of MRSA Isolates

**Figure 1:**
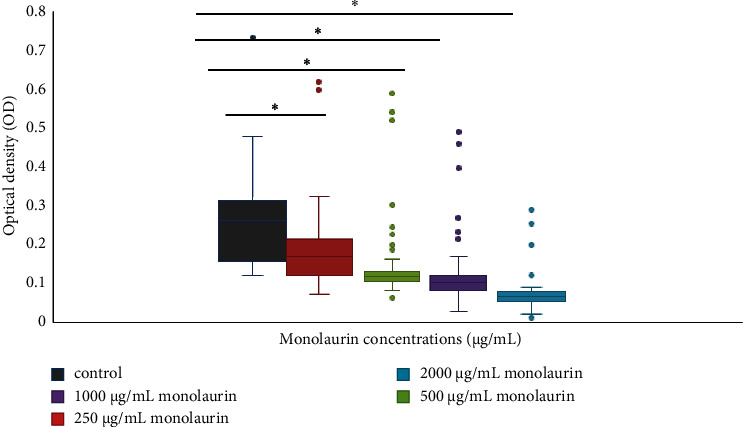
The optical density (OD) of biofilms formed upon exposure to varying concentrations of monolaurin by MRSA isolates (*n* = 103). The experiment was performed in triplicates. Grey color indicates control (OD of MRSA before treatment). Red color indicates OD after treatment with 250 *μ*g/mL of monolaurin. Green color indicates OD after treatment with 500 *μ*g/mL of monolaurin. Violet color indicates OD after treatment with 1000 *μ*g/mL of monolaurin. Blue color indicates OD after treatment with 2000 *μ*g/mL of monolaurin. ∗Significant difference at *p* value <0.05.

Investigating the possible use of monolaurin in the destruction of biofilms was a key objective in the treatment of biofilms. The ability of monolaurin to eradicate biofilms established by MRSA isolates is illustrated in Table 2. The optical density (OD) measurements of the treatment provided evidence that monolaurin effectively inhibited the preformed biofilms across all strains examined, as depicted in Figure 1. The IC_50_ (range) of monolaurin was 379.3 *μ*g/mL (280.1–515.6 *μ*g/mL) against MRSA isolates.

#### 3.6.2. Monolaurin Activity against Biofilm Formation of MRSA

**Figure 2:**
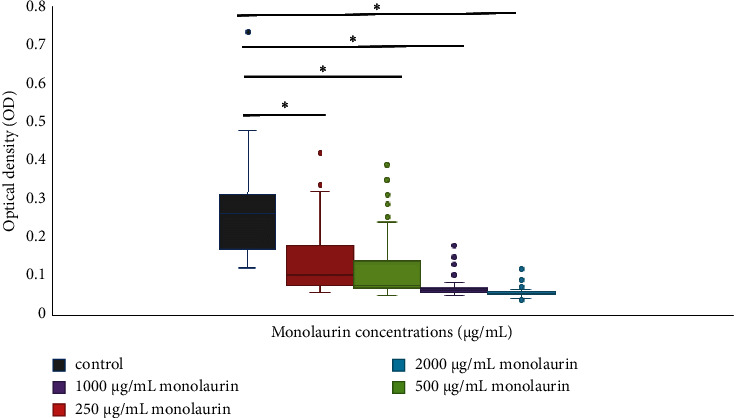
The optical density (OD) of biofilms developed following exposure to different concentrations of monolaurin by MRSA isolates (*n* = 103). The experiment was performed in triplicates. Grey color indicates control (OD of MRSA before treatment). Red color indicates OD after treatment with 250 *μ*g/mL of monolaurin. Green color indicates OD after treatment with 500 *μ*g/mL of monolaurin. Violet color indicates OD after treatment with 1000 *μ*g/mL of monolaurin. Blue color indicates OD after treatment with 2000 *μ*g/mL of monolaurin. ∗Significant difference at *p* value <0.05.

As demonstrated in Table 3, monolaurin had a remarkable capacity to prevent the biofilm formation of all MRSA strains when compared to control cells cultured in monolaurin-free media. Optical density (OD) values of the treatment, as shown in Figure 2, convincingly demonstrated that monolaurin reduced biofilm development in each of the tested strains. The IC_50_ (range) of monolaurin was 203.6 *μ*g/mL (157.9–258.6 *μ*g/mL) against MRSA isolates.

### 3.7. Scanning Electron Microscope

**Figure 3:**
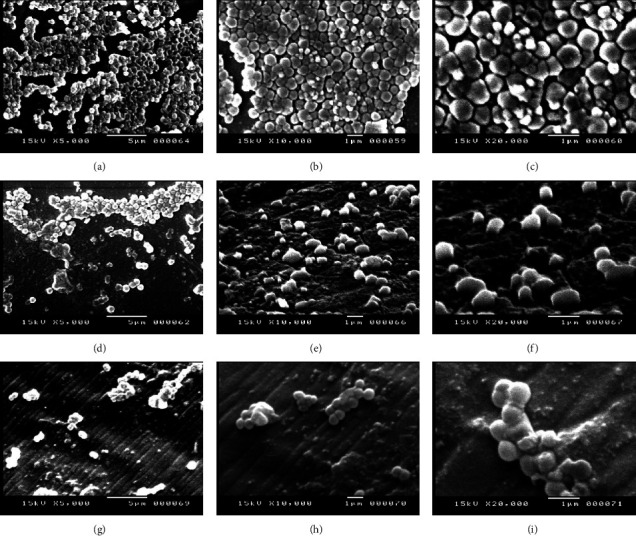
Scanning electron micrographs illustrating the eradication of MRSA isolate biofilms. MRSA biofilms depicted in the absence (a, b, c) and presence (d, e, f) of 1000 *μ*g/mL of monolaurin, and presence (g, h, i) of 2000 *μ*g/mL of monolaurin. Magnification levels: 5,000x (a, d, g); 10,000x (b, e, h); and 20,000x (c, f, i).

**Figure 4:**
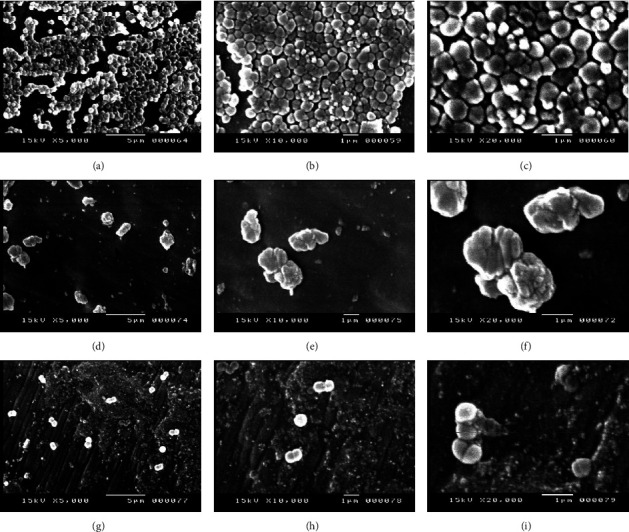
Scanning electron micrographs illustrating the inhibition of biofilm formation in MRSA isolates. MRSA biofilms observed in the absence (a, b, c) and presence (d, e, f) of 1000 *μ*g/mL monolaurin, and presence (g, h, i) of 2000 *μ*g/mL monolaurin. Magnification levels: 5,000x (a, d, g); 10,000x (b, e, h); and 20,000x (c, f, i).

Scanning electron microscopy (SEM) was used to confirm the effect of monolaurin on the eradication of MRSA biofilm. As can be seen in the SEM image, MRSA biofilms were clearly visible on a coverslip in the control group. When *S. aureus* biofilms were treated with monolaurin at concentrations of 1000 and 2000 *μ*g/mL, it was found that the density of biofilms had diminished (Figure 3).  Figure 4 shows the effect of monolaurin on the inhibition of biofilm produced by the tested isolate. In comparison to control (untreated cells), it was found that treating cells with monolaurin resulted in fewer cell attachments and decreased biofilm development at concentrations of 1000 and 2000 *μ*g/mL.

### 3.8. Conventional PCR

Genomic analysis was conducted on 59 MRSA strains identified as strong biofilm producers, isolated from different types of wound infections, with the aim of detecting the presence of the *icaA* and *icaD* genes. Out of the tested samples, 52 (88.14%) samples were *icaD* positive, while all isolates were negative for the *icaA* gene as illustrated in Supplementary Figure (SF-4). The distribution of *icaD* genes among different types of wound infection is illustrated in Supplementary Table (ST-7).

### 3.9. Effect of Monolaurin on Expression of *icaD* Gene among MRSA Strains

**Figure 5:**
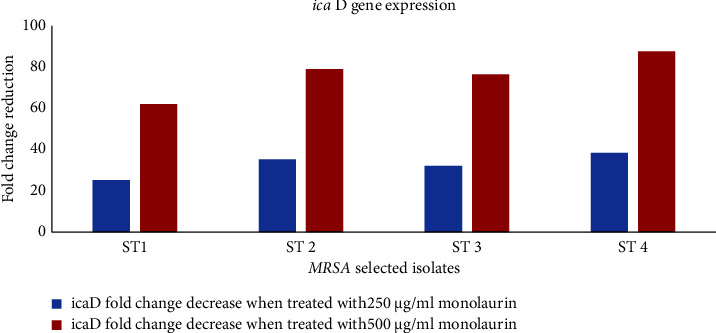
Effect of sub-MICs of monolaurin on expression of biofilm-related *icaD* gene among MRSA strains.

Quantitative real-time PCR was employed to assess the gene expression of *icaD* in samples treated with monolaurin at 250 *μ*g/mL and 500 *μ*g/mL concentrations. Four isolates were chosen for testing the activity of monolaurin on the gene expression of the *icaD* gene. Upon using 250 *μ*g/mL of monolaurin on the tested strains, a fold decrease in the expression of the *icaD* gene ranged from 25.26 to 38.44, while when using 500 *μ*g/mL of monolaurin, fold change ranged from 62.11 to 87.76 compared to the positive control samples (Figure 5).

## 4. Discussion

Methicillin-resistant *Staphylococcus aureus* (MRSA) is a common antibiotic-resistant organism contributing significantly to both community and hospital-associated infections. The fact that MRSA may develop biofilms on both biotic and abiotic surfaces adds complexity to the challenge posed by this organism. *Staphylococci* have been recognized for their ability to prompt biofilm-associated infections [30–32]. The resilience of established biofilms to various inhibitory techniques makes them particularly challenging to regulate or eradicate [33]. The development of effective antibiofilm strategies is crucial not only for enhanced treatment of biofilm-related diseases but also for mitigating the acceleration and distribution of antibiotic resistance in affected individuals [34]. Natural antibiofilm compounds have been discovered as a result of biofilm's increased antibiotic resistance rate [35]. Monolaurin, a naturally occurring substance frequently present in coconut oil, exhibits strong antibacterial properties. Our investigation was assumed to elucidate the potential impact of monolaurin on both biofilm development and preformed biofilms generated by the examined MRSA isolates.

In the present study, out of the 155 samples, 115 (74.19%) were positive for *S. aureus*. In 115 *S. aureus* samples, 103 (89.56%) were MRSA. This result was in alignment with the findings of Rehman et al. [36], who reported that 78.3% of the clinical isolates of *S. aureus* collected from tertiary care hospitals in Peshawar, Pakistan, were MRSA. Alreeme et al. [37] highlighted *Mycobacterium tuberculosis* and Methicillin-resistant *S. aureus* as the most detectable microorganisms associated with respiratory diseases during Hajj. Our study revealed also a heightened incidence of MRSA (37.9%) in patients with accidental wound infection compared to other types of infections. Correspondingly, Garoy et al. [38] found MRSA detection rates of 62.5%, 60%, 78.6%, and 87.5% in patients with surgical wounds, abscesses, and burns, respectively. El Amin and Faidah [39] identified MRSA as the most frequently isolated pathogen in wound infections. In addition, Alahmadi et al. [40] emphasized the rising issue of multidrug-resistant hospital-acquired MRSA in Saudi Arabia due to inappropriate antibiotic use.

The antibiogram of the studied MRSA strains revealed that linezolid and imipenem were the most effective antibiotic against *S. aureus* (2% and 3% resistance rates, respectively) followed by vancomycin (4.35% resistance rate) and chloramphenicol (13.9% resistance rate). MRSA showed complete resistance to amoxicillin/clavulanic acid, piperacillin/tazobactam, ampicillin/sulbactam, and cefoxitin (100%) and moderate resistance to tetracycline (64.07%), rifampicin (40.77%), ciprofloxacin and levofloxacin (38.8%), and gentamicin (37.86%). Similar results were obtained by Rehman et al. [36], who indicated that MRSA isolates had 100% resistance to penicillin and cefoxitin and low resistance to both chloramphenicol (14.46%) and linezolid (2.41%), respectively. In another study performed by Jafari-Sales et al. [41], they showed that MRSA isolated from tertiary care hospitals in the North Batinah region, Oman, were completely resistant to penicillin and highly resistant to amoxicillin/clavulanic acid (97.6%). Sannathimmappa et al. [42] reported a higher ciprofloxacin resistance (30.2%) among MRSA than observed in our study. Notably, our study identified each isolated MRSA strain as multidrug-resistant (MDR), consistent with observations made by Blomfeldt et al. [43] and Deyno et al. [44].

Due to the high prevalence of MDR bacteria, numerous researchers tried alternative strategies to assess the impact of nonantimicrobial agents on the growth of these pathogens. Investigations have explored the potential efficacy of substances such as essential oils, plant extracts, and certain food supplements [45–47]. Within the scope of our study, the MIC range for monolaurin against MRSA was determined to be 250–2000 *μ*g/mL. Comparable results were observed in a study conducted by Nitbani et al. [48], in which monolaurin was found to inhibit the growth of *S. aureus*, including MRSA, even at the lowest concentration tested (500 *μ*g/mL). Furthermore, when compared to standard strains *S. aureus* ATCC 25923 and ATCC 1885, monolaurin exhibited MICs of 100 and 250 *μ*g/mL, respectively [49, 50].

Drug combination therapy is a workable strategy, but it must be chosen carefully to prevent the emergence of resistance [51]. The FICi results for synergy confirmed the synergistic antibacterial activity of *β*-lactam and monolaurin antibiotics against MRSA isolates [52]. It has been demonstrated that combining many antimicrobials can reduce the dosages needed for each antimicrobial while increasing their antibacterial activity [53]. The effective elimination of *Staphylococcal* infection in patients is greatly restricted by biofilms as they protect bacteria from the action of both the host immune system and antibacterial medications [54]. Various methods exist for evaluating bacterial biofilm formation, with the microtiter plate biofilm formation assay acknowledged as a particularly effective technique. In our investigation, biofilm formation was assessed by the tissue culture plate (TCP) method, and the findings revealed that 57.28% of MRSA isolates showed strong biofilms, while only 42.72% of the isolates produced moderate biofilms. Our findings were in agreement with a research performed by Hosseini et al. [55], who reported that 52.9% and 45.3% of MRSA strains were strong and moderate biofilm producers, in respective order. Also, Awan et al. [56] found that all the tested strains of *S. aureus* produced biofilms showing 63.6% as strong biofilm producers and 36.4% as moderate biofilm producers. In contrast, Gaire et al. [57] reported divergent findings, indicating that among 27 *S. aureus* strains, only 1 (4%) isolate exhibited strong biofilm production, 19 (70%) produced weak biofilms, and 7 (26%) produced moderate biofilms. Consequently, it appeared that the strains in our investigation exhibited heightened virulence and a greater propensity for strong biofilm production, potentially attributed to variations in sample sources or differences in antibiotic administration practices in Egypt.

The biofilms formed by the 103 MRSA strains involved in this study were examined using varying concentrations of monolaurin, specifically at 250, 500, 1000, and 2000 *μ*g/mL. Employing the tissue culture plate (TCP) method, crystal violet staining was utilized to quantify the effectiveness of monolaurin in inhibiting the formation of biofilms and eradicating those established by MRSA isolates. The results demonstrated that monolaurin exhibited a dose-dependent inhibitory effect on MRSA biofilm formation and eradication. As depicted in Tables 2 and 3, an escalation in monolaurin concentration corresponded to an augmentation in the inhibitory activity against biofilm formation. These results concurred with those of Krislee et al. [47], who observed the efficient suppression of *Staphylococcus epidermidis* biofilms by monolaurin at concentrations ranging from 1 to 1.9 mg/mL. Furthermore, it has been reported that glycerol monolaurate, at a concentration of 1,822 *μ*M, exhibited complete removal of *S. aureus* biofilms [58]. Following Mochtar et al., the authors in [59] found that monolaurin can diminish *S. epidermidis* biofilm formation by 80% at 1,953 *μ*g/mL. Also, monolaurin can suppress the development of biofilms by decreasing bacterial cell's hydrophobicity and preventing bacterial cell's adherence [60].

Using SEM analysis, we discerned the inhibitory and antibiofilm effects of monolaurin on MRSA biofilm. In the control group, MRSA exhibited strong biofilm formation on the coverslip, characterized by aggregates and microcolonies. However, upon the introduction of 1000 and 2000 *μ*g/mL concentrations of monolaurin into the culture conditions, a distinguished reduction in both biofilm cell count and extracellular matrix was observed. Our results were in accordance with a study performed by Gil et al. [61]. They found that the absorbance values for coated wires with monolaurin were much lower than those for plain wires, indicating the antibiofilm activity of monolaurin-coated wires against MSSA, MRSA, and *S. epidermidis*. Furthermore, SEM examination of *S. epidermidis* treated with monolaurin at concentrations ranging from 1000 to 1953 *μ*g/mL revealed diminished cell attachments and reduced biofilm production, supporting the inhibitory potential of monolaurin against biofilm formation [59].

Polysaccharide intercellular adhesin [62], which is made of *β*−1,6-*N*-acetylglucosamine and is responsible for cell-to-cell adhesion, is expressed in *S. aureus* strains and regulates the formation of biofilms. The chromosomal intercellular adhesion (*ica*) locus, comprising the *icaADBC* structural genes and the *icaR* regulatory gene, encodes PIA. The *icaA* and *icaD* genes within this locus govern the synthesis of PIA, thereby regulating the biofilm-forming ability of *S. aureus* strains. This suggests the potential utility of the *ica* locus as a therapeutic target for biofilm-associated infections [63]. Through conventional PCR, we identified the presence of both *icaA* and *icaD* genes in our study. Surprisingly, none of the examined isolates carried the *icaA* gene, while the *icaD* gene was detected in 88.14% of the tested MRSA isolates. Our results were consistent with observations of Darwish and Asfour [64], who noted that the *icaA* gene was less prevalent compared to the *icaD* gene, and the presence of either gene did not necessarily correlate with biofilm formation.

Real-time PCR analysis of the expression of *icaD* following treatment with 250 *μ*g/mL and 500 *μ*g/mL of monolaurin revealed a significant downregulation of gene expression following monolaurin treatment by 25.26–38.44 and 62.11–87.76-fold change, respectively, in comparison with their resultant levels prior to treatment. Consequently, monolaurin interfered with the expression of a gene associated with biofilm production (*icaD*). Our observations were found to be compatible with those of Schlievert and Peterson [58] and Nitbani et al. [65], whose research similarly indicated that monolaurin disrupts biofilm formation, resulting in lower polysaccharide growth and reduced cell adhesion inside biofilms *in vitro*. These encouraging results suggest the potential utility of monolaurin as antibiofilm coatings on medical devices to reduce biofilm formation and infections. Despite promising *in vitro* results, the effectiveness of monolaurin against MRSA biofilms in clinical practice may face challenges related to formulation optimization. Formulating monolaurin into stable and bioavailable preparations suitable for clinical use may pose technical challenges due to its poor solubility in water.

## 5. Conclusion

In this study, we evaluated the antibacterial effectiveness and potential synergistic effects of combining monolaurin with *β*-lactam antibiotics against methicillin-resistant *Staphylococcus aureus* (MRSA) isolates. The results indicated that monolaurin exhibits significant antibacterial activity. Moreover, employing this combination may allow for the reduction of individual antibiotic dosages required and potentially diminish the development of antibiotic resistance. Our study revealed the favorable antibacterial efficacy of monolaurin against clinical strains of MRSA known for their biofilm-producing capabilities. In addition, the study demonstrated an efficient eradication of pre-existing biofilms. Furthermore, a striking correlation was found between elevated monolaurin concentrations and a concomitant decrease in *icaD* gene expression levels. Henceforth, we propose consistent monitoring of biofilm development in MRSA strains obtained from wound specimens, along with an assessment of their patterns of antimicrobial resistance. Such surveillance could facilitate the establishment of a comprehensive antimicrobial strategy aimed at promptly addressing wound infections. This study investigated the impact of varying concentrations of monolaurin (250, 500, 1000, and 2000 *μ*g/mL) on MRSA biofilms. While findings demonstrated dose-dependent inhibition of biofilm formation, it is imperative to evaluate the practical implications for clinical application. Factors such as monolaurin availability, manufacturing challenges, associated costs, and potential dose-dependent side effects must be considered to align *in vitro* findings with *in vivo* outcomes accurately. Thus, while the study contributes valuable insights into monolaurin's therapeutic potential against MRSA biofilms, further research is essential to assess its feasibility and effectiveness in real-world medical settings.

## Figures and Tables

**Table 1:** Minimum inhibitory concentration of monolaurin (*μ*g/mL) on MRSA isolates.

No. of MRSA isolates		MIC of monolaurin (*μ*g/mL)
No	%^*∗*^	
3	2.9	250
9	8.7	500
83	80.6	1000
8	7.8	2000

%^*∗*^Correlated with total number of MRSA isolates (no = 103). MIC, minimum inhibitory concentration.

**Table 2:** Effect of monolaurin on the preformed biofilm of MRSA *isolates*.

Concentration of monolaurin (*μ*g/mL)	% of inhibition (reduction in OD)	No. of isolates	%^*∗*^	*P* value^*∗∗*^
250	0–20	42	40.8	<0.001
	21–40	38	36.9	
	41–60	19	18.4	
	61–80	4	3.9	
	81–100	—	—	
Total	103		100	

500	0–20	24	23.3	<0.001
	21–40	19	18.4	
	41–60	29	28.2	
	61–80	28	27.2	
	81–100	3	2.9	
Total	103		100	

1000	0–20	5	4.9	<0.001
	21–40	24	23.3	
	41–60	29	28.2	
	61–80	30	29.1	
	81–100	15	14.5	
Total	103		100	

2000	0–20	—	—	<0.001
	21–40	6	5.8	
	41–60	13	12.6	
	61–80	56	54.4	
	81–100	28	27.2	
Total	103		100	

%^*∗*^Correlated with total no of resistant isolates (no = 103). ^*∗∗*^Significant difference at *P* value <0.05. OD, optical density.

**Table 3:** Effect of monolaurin on MRSA *isolates* biofilm formation.

Concentration of monolaurin (*μ*g/mL)	% of inhibition (reduction in OD)	No. of isolates	%^*∗*^	*P* value^*∗∗*^
250	0–20	27	26.2	<0.001
	21–40	14	13.6	
	41–60	25	24.3	
	61–80	33	32	
	81–100	4	3.9	
Total	103		100	

500	0–20	10	9.7	<0.001
	21–40	20	19.42	
	41–60	24	23.3	
	61–80	38	36.9	
	81–100	11	10.68	
Total	103		100	

1000	0–20	2	1.94	<0.001
		
	21–40	7	6.8	
	41–60	22	21.36	
	61–80	47	45.63	
	81–100	25	24.27	
Total	103		100	

2000	0–20	—	—	<0.001
	21–40	7	6.8	
	41–60	13	12.6	
	61–80	46	44.7	
	81–100	37	35.9	
Total	103		100	

%^*∗*^Correlated with total no of resistant isolates (no = 103). ^*∗∗*^Refer to *P* value which was <0.001 *P* values <0.05 refer to significant difference. OD, optical density.

## Data Availability

The data used to support the findings of this study are included within the article and its Supplementary Information file.

## Supplementary Materials

Supplementary Materials Supplementary Figure SF-1: the distribution pattern of MRSA isolates in accordance with wound infection types. Supplementary Figure SF-2: the antimicrobial resistance patterns of MRSA isolates (*n* = 103) isolated from wound infection. Supplementary Figure SF-3: the multidrug resistance index (MDRI) profiles of MRSA isolates to the tested antibiotics. Supplementary Figure SF-4: agarose gel electrophoresis of amplified product *icaD* gene generated by PCR. Supplementary Table ST-1: the cycling conditions of the primers during PCR reaction. Supplementary Tables ST-2 and ST-3: oligonucleotide primers and conditions for RT-PCR. Supplementary Tables ST-4, ST-5, and ST-6: the reduction of MICs of ampicillin, amoxicillin, and piperacillin, respectively, in combination with monolaurin (ML) at different concentrations. Supplementary Table ST-7: the distribution of *icaD* among tested MRSA isolates based on wound infection type.

## References

[1] Goudarzi M., Navidinia M. (2019). Overview perspective of bacterial strategies of resistance to biocides and antibiotics. *Archives of Clinical Infectious Diseases*.

[2] Goudarzi M., Navidinia M., Beiranvand E., Goudarzi H. (2018). Phenotypic and molecular characterization of methicillin-resistant *Staphylococcus aureus* clones carrying the Panton-Valentine leukocidin genes disseminating in Iranian hospitals. *Microbial Drug Resistance*.

[3] Brady R. A., Leid J. G., Calhoun J. H., Costerton J. W., Shirtliff M. E. (2008). Osteomyelitis and the role of biofilms in chronic infection. *FEMS Immunology and Medical Microbiology*.

[4] Taponen S., Pyörälä S. (2009). Coagulase-negative staphylococci as cause of bovine mastitis—not so different from *Staphylococcus aureus*?. *Veterinary Microbiology*.

[5] Cassat J. E., Lee C. Y., Smeltzer M. S. (2007). Investigation of biofilm formation in clinical isolates of *Staphylococcus aureus*. *Methicillin-resistant Staphylococcus aureus (MRSA) protocols*.

[6] Del Pozo J., Patel R. (2007). The challenge of treating biofilm‐associated bacterial infections. *Clinical Pharmacology & Therapeutics*.

[7] Vestby L. K., Grønseth T., Simm R., Nesse L. L. (2020). Bacterial biofilm and its role in the pathogenesis of disease. *Antibiotics*.

[8] Cámara M., Green W., MacPhee C. E. (2022). Economic significance of biofilms: a multidisciplinary and cross-sectoral challenge. *Npj Biofilms and Microbiomes*.

[9] Flemming H.-C., Wingender J., Szewzyk U., Steinberg P., Rice S. A., Kjelleberg S. (2016). Biofilms: an emergent form of bacterial life. *Nature Reviews Microbiology*.

[10] Flemming H., Wingender J. (2010). The biofilm matrix. *Nature Reviews Microbiology*.

[11] Fux C. A., Costerton J. W., Stewart P. S., Stoodley P. (2005). Survival strategies of infectious biofilms. *Trends in Microbiology*.

[12] Abdel-Maksoud M., El-Shokry M., Ismail G. (2016). Methicillin-resistant *Staphylococcus aureus* recovered from healthcare-and community-associated infections in Egypt. *International Journal of Bacteriology*.

[13] Qanash H., Alotaibi K., Aldarhami A. (2023). Effectiveness of oil-based nanoemulsions with molecular docking of its antimicrobial potential. *Bioresources*.

[14] Khan A. A., Sutherland J. B., Khan M. S., Althubiani A. S., Ahmad I. (2017). Applications of biofilm and quorum sensing inhibitors in food protection and safety. *Biofilms in Plant and Soil Health*.

[15] Lin Y.-C., Schlievert P. M., Anderson M. J. (2009). Glycerol monolaurate and dodecylglycerol effects on *Staphylococcus aureus* and toxic shock syndrome toxin-1 in vitro and in vivo. *PLoS One*.

[16] Weinstein M. P., Lewis J. S. (2020). The clinical and laboratory standards institute subcommittee on antimicrobial susceptibility testing: background, organization, functions, and processes. *Journal of Clinical Microbiology*.

[17] Magiorakos A.-P., Srinivasan A., Carey R. B. (2012). Multidrug-resistant, extensively drug-resistant and pandrug-resistant bacteria: an international expert proposal for interim standard definitions for acquired resistance. *Clinical Microbiology and Infection*.

[18] Fu X., Huang B., Feng F.-q. (2008). Shelf life of fresh noodles as affected by the food grade monolaurin microemulsion system. *Journal of Food Process Engineering*.

[19] Albano M., Karau M. J., Schuetz A. N., Patel R. (2020). Comparison of agar dilution to broth microdilution for testing in vitro activity of cefiderocol against gram-negative bacilli. *Journal of Clinical Microbiology*.

[20] Xu W., Zhou Q., Liu J. (2022). In vitro study of the interaction of gentamicin with ceftriaxone and azithromycin against neisseria gonorrhoeae using agar dilution method. *Antibiotics*.

[21] Manandhar S., Singh A., Varma A., Pandey S., Shrivastava N. (2018). Evaluation of methods to detect in vitro biofilm formation by staphylococcal clinical isolates. *BMC Research Notes*.

[22] Mathur T., Singhal S., Khan S., Upadhyay D., Fatma T., Rattan A. (2006). Detection of biofilm formation among the clinical isolates of staphylococci: an evaluation of three different screening methods. *Indian Journal of Medical Microbiology*.

[23] Manandhar S., Singh A., Varma A., Pandey S., Shrivastava N. (2018). Biofilm producing clinical *Staphylococcus aureus* isolates augmented prevalence of antibiotic resistant cases in tertiary care hospitals of Nepal. *Frontiers in Microbiology*.

[24] Bhandari S., Khadayat K., Poudel S. (2021). Phytochemical analysis of medicinal plants of Nepal and their antibacterial and antibiofilm activities against uropathogenic *Escherichia coli*. *BMC Complementary Medicine and Therapies*.

[25] Basco L. K., Ringwald P. (2003). In vitro activities of piperaquine and other 4-aminoquinolines against clinical isolates of Plasmodium falciparum in Cameroon. *Antimicrobial Agents and Chemotherapy*.

[26] Barakat H. S., Kassem M. A., El-Khordagui L. K., Khalafallah N. M. (2014). Vancomycin-eluting niosomes: a new approach to the inhibition of staphylococcal biofilm on abiotic surfaces. *American Association of Pharmaceutical Scientists*.

[27] Yan X., Gu S., Shi Y., Cui X., Wen S., Ge J. (2017). The effect of emodin on *Staphylococcus aureus* strains in planktonic form and biofilm formation in vitro. *Archives of Microbiology*.

[28] Bagcigil A. F., Taponen S., Koort J., Bengtsson B., Myllyniemi A.-L., Pyörälä S. (2012). Genetic basis of penicillin resistance of *S. aureus* isolated in bovine mastitis. *Acta Veterinaria Scandinavica*.

[29] Yuan J. S., Reed A., Chen F., Stewart C. N. (2006). Statistical analysis of real-time PCR data. *BMC Bioinformatics*.

[30] Cascioferro S., Carbone D., Parrino B. (2021). Therapeutic strategies to counteract antibiotic resistance in MRSA biofilm‐associated infections. *Journal of Medicinal Chemistry*.

[31] Mader R., Damborg P., Amat J.-P. (2021). EU-JAMRAI building the European antimicrobial resistance surveillance network in veterinary medicine (EARS-Vet). *Euro Surveillance*.

[32] Lebeaux D., Chauhan A., Rendueles O., Beloin C. (2013). From in vitro to in vivo models of bacterial biofilm-related infections. *Pathogens*.

[33] Oloketuyi S. F., Khan F. (2017). Inhibition strategies of Listeria monocytogenes biofilms—current knowledge and future outlooks. *Journal of Basic Microbiology*.

[34] Savage V. J., Chopra I., O’Neill A. J. (2013). *Staphylococcus aureus* biofilms promote horizontal transfer of antibiotic resistance. *Antimicrobial Agents and Chemotherapy*.

[35] Mishra R., Panda A. K., De Mandal S., Shakeel M., Bisht S. S., Khan J. (2020). Natural anti-biofilm agents: strategies to control biofilm-forming pathogens. *Frontiers in Microbiology*.

[36] Rehman L. U., Khan A. A., Afridi P., Rehman S. U., Wajahat M., Khan F. (2022). Prevalence and antibiotic susceptibility of clinical staphylococcus aureus isolates in various specimens collected from a tertiary care hospital, Hayatabad, Peshawar, Pakistan.: antibiotic susceptibility of clinical staphylococcus aureus isolates in various specimens. *Pakistan Journal of Health Sciences*.

[37] Alreeme S., Bokhary H., Craig A. T. (2022). Transmission of antimicrobial resistant bacteria at the Hajj: a scoping review. *International Journal of Environmental Research and Public Health*.

[38] Garoy E. Y., Gebreab Y. B., Achila O. O. (2019). Methicillin-resistant *Staphylococcus aureus* (MRSA): prevalence and antimicrobial sensitivity pattern among patients—a multicenter study in Asmara, Eritrea. *The Canadian Journal of Infectious Diseases & Medical Microbiology*.

[39] El Amin N. M., Faidah H. S. (2012). Methicillin-resistant *Staphylococcus aureus* in the western region of Saudi Arabia: prevalence and antibiotic susceptibility pattern. *Annals of Saudi Medicine*.

[40] Alahmadi T. F., Alahmadey Z. Z., Elbanna K., Neyaz L. A., Ahmad I., Abulreesh H. H. (2023). The prevalence and clinical characteristics of multidrug-resistant hospital-acquired *Staphylococcus aureus* in medina, Saudi Arabia. *Journal of Pure and Applied Microbiology*.

[41] Jafari-Sales A., Farhadi F., Ezdiyadi M., Tarbiat-Nazloo D. (2018). Study of antibiotic resistance pattern in methicillin-resistant *Staphylococcus aureus* isolated from clinical samples of hospitals in Tabriz–Iran. *International Journal of Biomedicine and Public Health*.

[42] Sannathimmappa M. B., Nambiar V., Aravindakshan R., Al-Kasaby N. M. (2022). Patterns of methicillin-resistant *Staphylococcus aureus* (MRSA) strains isolated at a tertiary care hospital. *Journal of Datta Meghe Institute of Medical Sciences University*.

[43] Blomfeldt A., Larssen K., Moghen A. (2017). Emerging multidrug-resistant Bengal Bay clone ST772-MRSA-V in Norway: molecular epidemiology 2004–2014. *European Journal of Clinical Microbiology & Infectious Diseases*.

[44] Deyno S., Toma A., Worku M., Bekele M. (2017). Antimicrobial resistance profile of staphylococcus aureus isolates isolated from ear discharges of patients at University of Hawassa comprehensive specialized hospital. *BMC Pharmacology and Toxicology*.

[45] Mohamed A. A., Alotaibi B. M. (2023). Essential oils of some medicinal plants and their biological activities: a mini review. *Journal of Umm Al-Qura University for Applied Sciences*.

[46] Jesline A., John N. P., Narayanan P., Vani C., Murugan S. (2015). Antimicrobial activity of zinc and titanium dioxide nanoparticles against biofilm-producing methicillin-resistant *Staphylococcus aureus*. *Applied Nanoscience*.

[47] Krislee A., Fadly C., Nugrahaningsih D. A. A., Nuryastuti T., Nitbani F. O., Sholikhah E. N. (2019). The 1-monolaurin inhibit growth and eradicate the biofilm formed by clinical isolates of Staphylococcus epidermidis. *BMC Proceedings*.

[48] Nitbani F. O., Siswanta D., Sholikhah E. N., Fitriastuti D., Fitriastuti D. (2018). Synthesis and antibacterial activity 1-monolaurin. *Oriental Journal of Chemistry*.

[49] Sadiq S., Imran M., Habib H. (2016). Potential of monolaurin based food-grade nano-micelles loaded with nisin Z for synergistic antimicrobial action against *Staphylococcus aureus*. *LWT--Food Science and Technology*.

[50] Tajik H., Raeisi M., Razavi Rohani S. M. (2014). Effect of monolaurin alone and in combination with EDTA on viability of *Escherichia coli* and *Staphylococcus aureus* in culture media and iranian white cheese. *Journal of Food Quality and Hazards Control*.

[51] Hill J. A., Cowen L. E. (2015). Using combination therapy to thwart drug resistance. *Future Microbiology*.

[52] Ghany S. S. H. A. E., Ibrahem R. A., El-Gendy A. O., El-Baky R. M. A., Mustafa A., Azmy A. F. J. B. I. D. (2024). Novel synergistic interactions between monolaurin, a mono-acyl glycerol and *β* lactam antibiotics against *Staphylococcus aureus*: an in vitro study. *BMC Infectious Diseases*.

[53] Wang H., Niu Y., Pan J., Li Q., Lu R. (2020). Antibacterial effects of Lactobacillus acidophilus surface-layer protein in combination with nisin against *Staphylococcus aureus*. *LWT*.

[54] Davies D. (2003). Understanding biofilm resistance to antibacterial agents. *Nature Reviews Drug Discovery*.

[55] Hosseini M., Shapouri Moghaddam A., Derakhshan S. (2020). Correlation between biofilm formation and antibiotic resistance in MRSA and MSSA isolated from clinical samples in Iran: a systematic review and meta-analysis. *Microbial Drug Resistance*.

[56] Awan A. B., Arshad M. M., Haque A., Haque A. (2022). Level of biofilm production by *Staphylococcus aureus* isolates is critical for resistance against most but not all antimicrobial drugs. *Pakistan Journal of Medical Sciences*.

[57] Gaire U., Thapa Shrestha U., Adhikari S. (2021). Antibiotic susceptibility, biofilm production, and detection of mec A gene among *Staphylococcus aureus* isolates from different clinical specimens. *Diseases*.

[58] Schlievert P. M., Peterson M. L. (2012). Glycerol monolaurate antibacterial activity in broth and biofilm cultures. *PLoS One*.

[59] Mochtar C. F., Sholikhah E. N., Agung D. A. (2021). Inhibitory and eradication activities of 1-monolaurin as anti-biofilm on monospecies and polymicrobial of Staphylococcus epidermidis and *Candida tropicalis*. *International Journal of Pharmaceutical Research*.

[60] Ham Y., Kim T.-J. (2016). Inhibitory activity of monoacylglycerols on biofilm formation in *Aeromonas hydrophila*, *Streptococcus mutans*, *Xanthomonas oryzae*, and *Yersinia enterocolitica*. *SpringerPlus*.

[61] Gil D., Shuvaev S., Frank-Kamenetskii A., Reukov V., Gross C., Vertegel A. (2017). Novel antibacterial coating on orthopedic wires to eliminate pin tract infections. *Antimicrobial Agents and Chemotherapy*.

[62] Antonelli M., De Pascale G., Ranieri V. M. (2012). Comparison of triple-lumen central venous catheters impregnated with silver nanoparticles (AgTive®) vs conventional catheters in intensive care unit patients. *Journal of Hospital Infection*.

[63] Oliveira A., Cunha M. d. L. R. (2010). Comparison of methods for the detection of biofilm production in coagulase-negative staphylococci. *BMC Research Notes*.

[64] Darwish S. F., Asfour H. A. (2013). Investigation of biofilm forming ability in Staphylococci causing bovine mastitis using phenotypic and genotypic assays. *The Scientific World Journal*.

[65] Nitbani F. O., Tjitda P. J. P., Nitti F., Jumina J., Detha A. I. R. (2022). Antimicrobial properties of lauric acid and monolaurin in virgin coconut oil: a review. *Chemical-Biological Engineering Reviews*.

