# Supplementary material for: Antimicrobial and Antibiofilm Activity of Monolaurin against Methicillin-Resistant Staphylococcus aureus Isolated from Wound Infections
Source: Int J Microbiol. 2024 Aug 2;2024:7518368. doi: 10.1155/2024/7518368 (PMC11315973; doi:10.1155/2024/7518368)
Supplement: Supplementary Materials — Supplementary Figure SF-1: the distribution pattern of MRSA isolates in accordance with wound infection types. Supplementary Figure SF-2: the antimicrobial resistance patterns of MRSA isolates (n = 103) isolated from wound infection. Supplementary Figure SF-3: the multidrug resistance index (MDRI) profiles of MRSA isolates to the tested antibiotics. Supplementary Figure SF-4: agarose gel electrophoresis of amplified product icaD gene generated by PCR. Supplementary Table ST-1: the cycling conditions of the primers during PCR reaction. Supplementary Tables ST-2 and ST-3: oligonucleotide primers and conditions for RT-PCR. Supplementary Tables ST-4, ST-5, and ST-6: the reduction of MICs of ampicillin, amoxicillin, and piperacillin, respectively, in combination with monolaurin (ML) at different concentrations. Supplementary Table ST-7: the distribution of icaD among tested MRSA isolates based on wound infection type. [file 7518368.f1.docx]

**Supplementary materials**

- **Supplementary Figures (SF)**


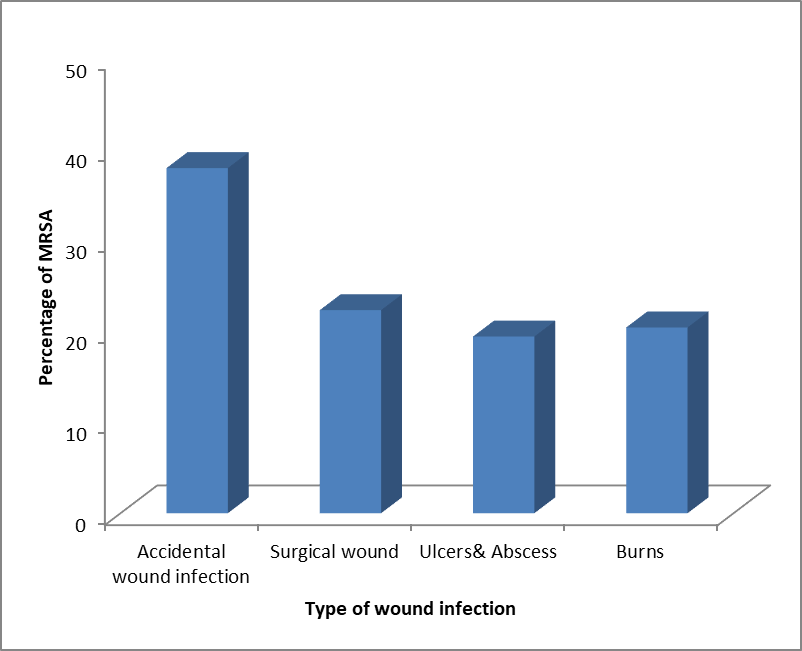


**SF-1: Distribution pattern of MRSA isolates in accordance with wound infection type.**

**
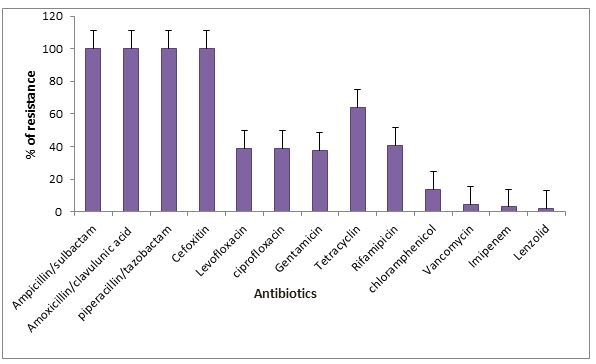
**

**SF-2: Antimicrobial resistance patterns of MRSA isolates (n = 103) isolated from wound infection. Error bar represents the standard error.**


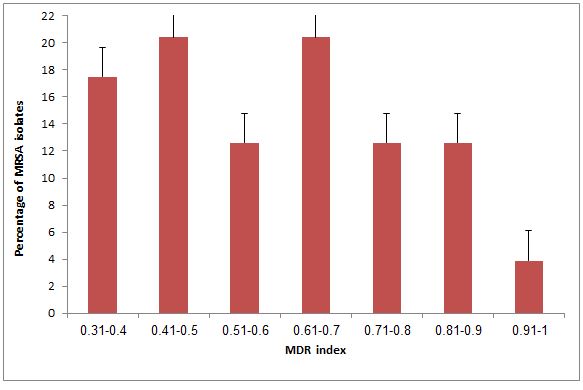


**SF-3: Multidrug resistance index (MDRI) profiles of MRSA isolates to the tested antibiotics. Error bar represents the standard error.**


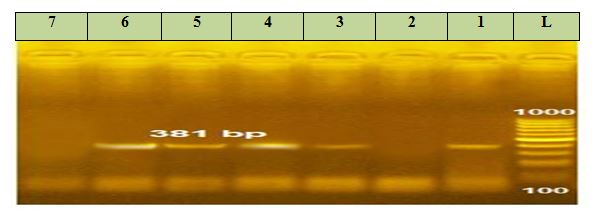


**SF-4: Agarose gel electrophoresis of amplified product *icaD* gene generated by PCR. Lane (L): molecular size marker (100bps), Lane 1, 3, 4, 5, 6: *icaD* positive samples and Lane 2, 7: *icaD* negative sample.**

**Supplementary Tables (ST)**

**.Cycling conditions of the primers during PCR reaction: ST-1**

| **Gene** | **Conditions** | | | | | | **Reference** |
| --- | --- | --- | --- | --- | --- | --- | --- |
|  | **Primary Denaturation** | **Secondary Denaturation** | **Annealing** | **Extension** | **No. of cycles** | **Final extension** |  |
| ***icaA*** | 94˚C  5 min. | 94˚C  30 sec. | 49˚C  1 min. | 72˚C  1 min. | 35 | 72˚C  12 min. | **[1]** |
| ***icaD*** | 94˚C  5 min. | 94˚C  30 sec. | 49˚C  40 sec. | 72˚C  40 sec. | 35 | 72˚C  7 min. |  |

**. Oligonucleotide primers and probes used in SYBR Green real time PCR : ST-2**

| **Gene** | **Primer sequence**  **(5'-3')** | **Product size**  **(bp)** | **Reference** |
| --- | --- | --- | --- |
| ***icaD*** | **F** AAA CGT AAG AGA GGT GG | **381** | **[2]** |
|  | **R** GGC AAT ATG ATC AAG ATA |  |  |
| ***16S rRNA*** | **F** CCTATAAGACTGGGATAACTTCGGG | **791** | **[3]** |
|  | **R** CTTTGAGTTTCAACCTTGCGGTCG |  |  |

**ST-3: Condition for real time PCR:**

| **Target gene** | **Reverse transcription** | **Primary**  **denaturation** | **Amplification (40 cycles)** | | | **Dissociation curve**  **(1 cycle)** | | |
| --- | --- | --- | --- | --- | --- | --- | --- | --- |
|  |  |  | **Secondary denaturation** | **Annealing** | **Extension** | **Secondary denaturation** | **Annealing** | **Final denaturation** |
| ***icaD*** | 50˚C  30 min. | 94˚C  15 min. | 94˚C  15 sec. | 49˚C  30 sec. | 72˚C  40 sec. | 94˚C  1 min. | 49˚C  1 min. | 94˚C  1 min. |
| ***16S rRNA*** |  |  |  | 55˚C  40 sec. |  |  | 55˚C  1 min. |  |

**ST-4: Reduction in minimum inhibitory concentration (MICs) of ampicillin combined with Monolaurin (ML) at different concentrations.**

| **Monolaurin**  **(Drug A)** | **MIC(µg/ml)** | | **No. of isolates** | **%*** | **Fractional inhibitory concentration index**  **(FICI)**** | **Behavior** | | **P value***** |
| --- | --- | --- | --- | --- | --- | --- | --- | --- |
|  | **Ampicillin**  **(Drug B)** | **MIC of ampicillin in presence of monolaurin** |  |  |  |  |  |  |
| 250 | 8 | 1 | 18 | 17.48 | 0.125 | **Synergistic** | | **<0.001** |
|  |  | 2 | 20 | 19.42 | 0.25 | **Synergistic** | |  |
|  | 16 | 1 | 16 | 15.5 | 0.0625 | **Synergistic** | |  |
|  |  | 4 | 16 | 15.5 | 0.25 | **Synergistic** | |  |
|  | 32 | 1 | 22 | 21.4 | 0.03125 | **Synergistic** | |  |
|  |  | 4 | 11 | 10.7 | 0.125 | **Synergistic** | |  |
| **Total no. of isolates** | **103** | | | | | | | |
| 500 | 8 | 0.5 | 27 | 26.2 | 0.0625 | | **Synergistic** | **<0.001** |
|  |  | 2 | 11 | 10.7 | 0.25 | | **Synergistic** |  |
|  | 16 | 0.5 | 28 | 27.1 | 0.03125 | | **Synergistic** |  |
|  |  | 2 | 4 | 3.9 | 0.125 | | **Synergistic** |  |
|  | 32 | 0.5 | 22 | 21.4 | 0.015625 | | **Synergistic** |  |
|  |  | 2 | 11 | 10.7 | 0.0625 | | **Synergistic** |  |
| **Total no. of isolates** | **103** | | | | | | | |

**^%* was correlated to total no of resistant isolates (no=103),^** ^**^**^total synergistic (FICI ≤ 0.5), partial synergistic (0.5 < FICI ≤ 0.75), or indifferent (0.75 < FICI ≤ 2 and antagonistic (FICI > 2)^**

**^***significant difference at P value < 0.05^**

**ST-5: Reduction in minimum inhibitory concentration (MICs) of amoxicillin combined with Monolaurin (ML) at different concentrations.**

| **Monolaurin**  **(Drug A)** | **MIC(µg/ml)** | | **No. of isolates** | **%*** | **Fractional inhibitory concentration index**  **(FICI)**** | **Behavior** | **P value***** |
| --- | --- | --- | --- | --- | --- | --- | --- |
|  | **Amoxicillin**  **(Drug B)** | **MIC of amoxicillin in presence of monolaurin** |  |  |  |  |  |
| 250 | 32 | 0.5 | 6 | 5.8 | 0.015625 | **Synergistic** | **<0.001** |
|  |  | 4 | 11 | 10.7 | 0.125 | **Synergistic** |  |
|  |  | 8 | 12 | 11.7 | 0.25 | **Synergistic** |  |
|  | 64 | 0.5 | 32 | 31 | 0.0078 | **Synergistic** |  |
|  |  | 2 | 5 | 4.9 | 0.03125 | **Synergistic** |  |
|  |  | 4 | 16 | 15.5 | 0.0625 | **Synergistic** |  |
|  |  | 8 | 11 | 10.7 | 0.125 | **Synergistic** |  |
|  | 128 | 1 | 6 | 5.8 | 0.0078 | **Synergistic** |  |
|  |  | 8 | 4 | 3.9 | 0.0625 | **Synergistic** |  |
| **Total no. of isolates** | **103** | | | | | | |
| 500 | 32 | 0.5 | 23 | 22.3 | 0.015625 | Synergistic | **<0.001** |
|  |  | 4 | 6 | 5.8 | 0.125 | Synergistic |  |
|  | 64 | 0.5 | 53 | 51.5 | 0.0078 | Synergistic |  |
|  |  | 1 | 5 | 4.9 | 0.015625 | Synergistic |  |
|  |  | 4 | 6 | 5.8 | 0.0625 | Synergistic |  |
|  | 128 | 0.5 | 10 | 9.7 | 0.0039 | Synergistic |  |
| **Total no. of isolates** | **103** | | | | | | |

**^%* was correlated to total no of resistant isolates (no=103),^** ^**^**^total synergistic (FICI ≤ 0.5), partial synergistic (0.5 < FICI ≤ 0.75), or indifferent (0.75 < FICI ≤ 2 and antagonistic (FICI > 2)^**

**^***significant difference at P value < 0.05^**

**ST-6: Reduction in minimum inhibitory concentration (MICs) of piperacillin combined with Monolaurin (ML) at different concentrations.**

| **Monolaurin**  **(Drug A)** | **MIC(µg/ml)** | | | **No. of isolates** | **%*** | **Fractional inhibitory concentration index**  **(FICI)**** | **Behavior** | **P value***** |
| --- | --- | --- | --- | --- | --- | --- | --- | --- |
|  | **piperacillin**  **(Drug B)** | **MIC of piperacillin in presence of monolaurin** | |  |  |  |  |  |
| 250 | 16 | 4 | | 2 | 1.9 | 0.25 | **Synergistic** | **<0.001** |
|  |  | 8 | | 18 | 17.5 | 0.125 | **Synergistic** |  |
|  | 64 | 8 | | 9 | 8.7 | 0.0625 | **Synergistic** |  |
|  |  | 16 | | 12 | 11.7 | 0.25 | **Synergistic** |  |
|  | 128 | 8 | | 12 | 11.7 | 0.03125 | **Synergistic** |  |
|  |  | 16 | | 20 | 19.4 | 0.125 | **Synergistic** |  |
|  |  | 32 | | 16 | 15.5 | 0.25 | **Synergistic** |  |
|  | 256 | 32 | | 14 | 13.6 | 0.125 | **Synergistic** |  |
| **Total no. of isolates** | **103** | | | | | | | |
| 500 | 16 | 0.5 | 26 | | 25.2 | 0.0078 | **Synergistic** | **<0.001** |
|  |  | 1 | 2 | | 1.9 | 0.0625 | **Synergistic** |  |
|  | 64 | 0.5 | 5 | | 4.8 | 0.0039 | **Synergistic** |  |
|  |  | 1 | 4 | | 3.9 | 0. 015625 | **Synergistic** |  |
|  | 128 | 1 | 16 | | 15.5 | 0.0078 | **Synergistic** |  |
|  |  | 8 | 12 | | 11.7 | 0.0625 | **Synergistic** |  |
|  |  | 16 | 12 | | 11.7 | 0.125 | **Synergistic** |  |
|  | 256 | 0.5 | 12 | | 11.7 | 0.00195 | **Synergistic** |  |
|  |  | 1 | 6 | | 5.8 | 0.0039 | **Synergistic** |  |
|  |  | 8 | 8 | | 7.8 | 0.03125 | **Synergistic** |  |
| **Total no. of isolates** | **103** | | | | | | | |

**^%* was correlated to total no of resistant isolates (no=103),^** ^**^**^total synergistic (FICI ≤ 0.5), partial synergistic (0.5 < FICI ≤ 0.75), or indifferent (0.75 < FICI ≤ 2 and antagonistic (FICI > 2)^**

**^***significant difference at P value < 0.05^**

**ST-7: Distribution of *icaD* among tested MRSA isolates based of wound infection type.**

| **Type of infection** | | **No of *icaD* positive isolates** | |
| --- | --- | --- | --- |
|  |  | **NO** | **%*** |
| **Wound infection** | Accidental wound infection | 23 | **39** |
|  | Ulcers& Abscess | 19 | **32.2** |
|  | Surgical wound | 8 | **13.55** |
|  | Burns | 2 | **3.39** |
| **Total** | | 52 | **88.14** |

**^%* was relative to tested no. of^ *^MRSA^* ^isolates (no=59)^**

**References:**

1. A. Ciftci, A. Findik, E. E. Onuk, S. Savasan, “Detection of methicillin resistance and slime factor production of Staphylococcus aureus in bovine mastitis,” *Brazilian Journal of Microbiology*, vol. 40, pp. 254-261, 2009.

2. A. F. Bagcigil, S. Taponen, J. Koort, B. Bengtsson, A.-L. Myllyniemi, S. Pyörälä, “Genetic basis of penicillin resistance of S. aureus isolated in bovine mastitis,” *Acta Veterinaria Scandinavica*, vol. 54, no. 1, pp. 1-7, 2012.

3. W. J. Mason, J. S. Blevins, K. Beenken, N. Wibowo, N. Ojha, M. S. Smeltzer, “Multiplex PCR protocol for the diagnosis of staphylococcal infection,” *Journal of clinical microbiology*, vol. 39, no. 9, pp. 3332-3338, 2001.
